# *Bifidobacterium animalis* subsp. *Lactis* BX-BC08 modulates gut microbiota and secretes alpha-Ketoglutaric acid to alleviate MC903-induced atopic dermatitis

**DOI:** 10.1186/s12967-025-06769-9

**Published:** 2025-07-10

**Authors:** Jiamin Zhao, Ling Kui, Jinqun Huang, Jie Deng, Lingjun Liu, Chenwei Zhu, Yanqiang Shi, Chengyi Li, Yue Xiao, Jinshi Yu, Qing Li, Bin Yang, Bingfeng Leng, Hung Chan

**Affiliations:** 1 Dermatology Hospital, Southern Medical University, Guangzhou, China; 2 Hong Kong Rising Biotechnology Co. Limited, Hong Kong, China; 3 Dermatology Department & Medical Cosmetology Department, Shenzhen Qianhai Shekou Free Trade Zone Hospital, Shenzhen, China; 4 Shenzhen Beichen Biotech Co., Ltd, Shenzhen, China; 5 Department of Dermatology and Venereology, West China Hospital, Sichuan University, Chengdu, Sichuan China; 6 University of California San Diego, San Diego. La Jolla, CA USA

**Keywords:** Atopic dermatitis (AD), alpha-Ketoglutaric acid (AKG), Probiotics, *Bifidobacterium*, BX-BC08, Skin-gut axis

## Abstract

**Objective:**

*Bifidobacterium* is known to be depleted in patients with atopic dermatitis (AD). This study aims to investigate the potential prophylactic effects of *Bifidobacterium animalis* subsp. *lactis* BX-BC08 (*B. lactis* BX-BC08) in a murine model of AD.

**Design:**

The immunosuppressive and anti-inflammatory effects of BX-BC08 were evaluated in a MC903-induced AD mouse model. Gut microbiota composition was analyzed by metagenomic sequencing, while high-performance liquid chromatography-mass spectrometry (HPLC-MS) was employed to identify anti-inflammatory molecules produced by *B. lactis* BX-BC08.

**Results:**

BX-BC08 significantly attenuated pro-inflammatory responses, scaling and swelling in the MC903-induced AD like murine model compared to controls. Fecal microbial profiling revealed an enrichment of probiotics and a reduction of pro-inflammatory bacteria in BX-BC08 treated mice. Metabolic analysis of BX-BC08 bacteria culture supernatant and treated mice identified a significant enrichment of alpha-Ketoglutaric acid (AKG). Functional validation in the murine AD model demonstrated that AKG strongly suppressed T helper 2 (Th2)-driven pro-inflammatory responses.

**Conclusion:**

BX-BC08 mitigates AD-like inflammation by producing the anti-inflammatory metabolite AKG. BX-BC08 could serve as a novel prophylactic agent for AD prevention.

**Supplementary Information:**

The online version contains supplementary material available at 10.1186/s12967-025-06769-9.

## Introduction

Atopic dermatitis (AD) is a chronic, relapsing inflammatory skin disease characterized by a strong genetic predisposition and intense pruritus, significantly impacting patient’s quality of life. Clinically, AD presents with recurrent eczema-like lesions and severe itching, often leading to considerable discomfort. Globally, AD has become increasingly prevalent, with epidemiological studies reporting an incidence of approximately 20% in pediatric populations and 10% in adults (Langan, Irvine, & Weidinger [1]; Nutten [2].

The pathogenesis of AD is multifactorial, driven by a complex interplay of genetic susceptibility, immune dysregulation, and environmental triggers (Irvine, McLean, & Leung [3]; K. E. Kim, Cho, & Park [4]). These pathogenic factors culminate in a biphasic T-helper cell imbalance, characterized by an initial predominance of Th2-polarized responses. The overproduction of Th2-associated cytokines-including interleukin-4 (IL-4), interleukin-13 (IL-13), interleukin-25 (IL-25), and thymic stromal lymphopoietin (TSLP)-is accompanied by the recruitment of immune cells [5], further driving the induction of allergic sensitization.

Although current treatments such as glucocorticoids and antihistamines provide reduction in pro-inflammatory responses [6], the side effects associated with these therapies (Schäcke, Döcke, & Asadullah [7]; Simons [8], remain unresolved. Therefore, alternative therapies that offer similar efficacy with fewer side effects are urgently needed.

Emerging evidence suggests that gut microbiota play a crucial role in the pathogenesis of AD [9]. Clinical observations have shown a significant depletion of *Bifidobacterium* populations in AD patients [10], leading to impairments in intestinal barrier integrity through the degradation of tight junction proteins [11]. This disruption subsequently amplifies Th2 responses (Salem, Ramser, Isham, & Ghannoum [12]), and exacerbates AD symptomatology [13]. Restoring the gut microbiota holds promising potential as a preventive treatment for the onset of AD [14, 15]; J. H. Kim, Kim, & Kim [16]. *Bifidobacterium* acts as a key probiotic genus, regulating host physiology and disease processes through microbiota modulation (Turroni et al., 2014). It is known to suppress the abundance of pathogenic microorganisms (Arboleya, Watkins, Stanton, & Ross, 2016) (Belkaid & Hand, 2014) by producing acetate and lactate (Pokusaeva, Fitzgerald, & van Sinderen [17]), and secreting bacteriocins (O’Shea, Cotter, Stanton, Ross, & Hill, 2012). However, the precise role of *Bifidobacterium* in contributing to skin diseases, particularly in modulating the Th2 responses, remains elusive.

In this study, we show that *B. lactis* BX-BC08 alleviates symptoms in AD-like mouse models by reshaping the gut microbiota and producing the anti-inflammatory metabolite AKG.

## Materials and methods

### Animal experiment

All animal experiments conducted were approved by the Southern Medical University, Institutional Animal Care and Use Committee (IACUC number: 2024F818). Female BALB/c mice (6–8 weeks old) were housed under under specific pathogen-free (SPF) conditions and used to establish AD models. After a three-day acclimatization period, the mice were assigned to three experimental groups using a randomization procedure to ensure unbiased group allocation. Both the vehicle control (nc group) and the brain heart infusion (BHI) group were administered BHI broth, and the BX-BC08 group was supplemented with *B*. *lactis* BX-BC08. The bacterial suspension was prepared by culturing *B. lactis* BX-BC08 in BHI broth for 24 h at 37 °C under anaerobic conditions. The culture was then diluted in sterile BHI medium to achieve a final concentration of 1 × 10^8^ colony-forming units (CFU) per 200 µL. Each mouse was administered 200 µL of the designated treatment daily via oral gavage for 21 consecutive days.

### MC903-Induced atopic dermatitis model

The MC903-induced AD model was established following a previously described protocol [18]. Mice in the BHI and BX-BC08 groups received daily topical applications of 100 µM MC903 (MedChemExpress), prepared by sequential dissolution in dimethyl sulfoxide (DMSO), followed by a 240-fold dilution in absolute ethanol. Ten microliters of the MC903 solution were applied bilaterally to the inner auricular surface using a micropipette, beginning on day 7 after gavage initiation and continuing for 14 consecutive days. Nc group received an equivalent volume of the ethanol vehicle alone. Dermatological assessments, including erythema severity, edema formation, and excoriation scores, were conducted daily using a modified version of the SCORing Atopic Dermatitis (SCORAD) index [19]. Prior to euthanasia, mouse feces were collected. The mice were then anesthetized, euthanized, and their blood and ear tissue were harvested for downstream analyses.

### α-ketoglutarate administration in an MC903-Induced atopic dermatitis model

BALB/c mice (6-8 weeks old) were provided with a 2% AKG solution or sterile water in their drinking water prior to MC903-induced ear inflammation. The designated treatment was administered for 21 consecutive days.

### Bacterial strains and culture conditions

BX-BC08 was isolated from traditional fermented yogurt sourced from Inner Mongolia households using a standardized protocol (Milani et al., 2017). Specifically, ten grams of yogurt homogenate was serially diluted in 90 mL sterile phosphate-buffered saline (PBS, pH 6.8). Subsequently, 0.1mL of the diluted sample was spread onto selective reinforced clostridial medium (RCM) supplemented with 0.05% (w/v) L-cysteine hydrochloride (Sigma-Aldrich). The inoculated plates were incubated anaerobically (AnaeroPack System, Mitsubishi Gas Chemical) at 37 °C for 72 h. Presumptive *Bifidobacterium* colonies were selected and identified through Gram staining (Gram-positive, irregular rods). Gram-positive strains were identified and further sub-cultured by streaking on RCM agar for 2-3 passages to ensure purity (Turroni et al., 2014). For experimental administration, the validated strain was cultured in pre-reduced BHI broth under anaerobic conditions (10% H_2_, 10% CO_2_, 80% N_2_) at 37 °C. Bacterial suspensions were centrifuged (3,000 ×g, 10 min, 4 °C), washed twice with sterile BHI, and adjusted to 1 × 10^8^ CFU/mL using optical density calibration curves (OD 600 = 0.8 ± 0.05) prior to gavage (Pokusaeva, Fitzgerald, & van Sinderen [17]).

### Histological analysis

Following euthanasia via isoflurane anesthesia (4% vol/vol induction, 2% maintenance), bilateral auricular tissues were surgically excised and immersion-fixed in 4% paraformaldehyde (Sigma-Aldrich) for 24 h at 4 °C. Fixed specimens were paraffin-embedded (Paraplast Plus, McCormick Scientific), sectioned coronally at 5 μm thickness using a rotary microtome (Leica RM2235) and mounted on poly-L-lysine-coated slides. Tissue sections underwent standard hematoxylin and eosin (H&E) staining (Thermo Fisher Scientific) prior to bright-field microscopy evaluation (Olympus BX53) (Jensen, 2008). Histomorphometric assessment quantified one key inflammatory parameter: epidermal hyperplasia (vertical thickness from stratum basales to the stratum granulosum). Section thickness was verified using a digital micrometer (Mitutoyo, ± 0.5 μm accuracy).

### Assessment of dermatitis severity

Cutaneous inflammation severity was evaluated macroscopically in a blinded manner using an established scoring system (Leung, Boguniewicz, Howell, Nomura, & Hamid, 2004). Lesion quantification incorporated four distinct clinical parameters: erythema intensity, edema thickness, scaling severity, and epidermal excoriation. Each parameter was graded on an ordinal scale: 0 = asymptomatic; 1 = mild manifestation (< 25% surface involvement); 2 = moderate presentation (25–50% involvement); 3 = severe pathology (> 50% involvement). The cumulative dermatitis score represented the arithmetic sum of individual parameter scores, with a maximum attainable score of 12 per specimen (Spergel & Paller, 2003).

### Real-Time quantitative reverse transcription PCR (qRT-PCR) for pro-Inflammatory cytokines

Murine auricular specimens were mechanically homogenized in *RNAsino Plus* preservation solution using two 5 mm ceramic homogenization beads in pre-chilled grinding tubes (Thermo Fisher Scientific, 2023). Total RNA was extracted, and complementary DNA (cDNA) was synthesized following standardized RT-qPCR protocols (Bustin et al., 2009). Target-specific primer pairs were designed for the amplification of Th2-associated cytokines (IL-4, IL-13) and epithelial-derived alarmins (IL-25, IL-33, TSLP), with thermal cycling parameters optimized using Primer-BLAST validated sequences (Ye et al., 2012). Relative quantification of inflammatory mediators was normalized to *Gapdh* expression, and fold-change calculations were performed using the 2^−ΔΔCt^ method (Livak & Schmittgen, 2001). Primer sequences are listed in Table S1.

### Metagenomic sequencing of BALB/c mice

To investigate the effects of *B. lactis* BX-BC08 on the gut microbiota, we performed metagenomic analysis of stool samples from BALB/c mice after three weeks of intragastric *Bifidobacterium* administration. Metagenomics sequencing generated raw data which underwent quality control to obtain clean data, followed by scaffolded contig (scaftig) assembly. Species and functional abundance data were used for abundance cluster analysis, non-metric Multidimensional Scaling (NMDS) analysis, and sample clustering to identify differences in species composition and functional profiles among samples. *P* value < 0.05 was considered statistically significant. Differential abundance analysis was performed with one-way analysis of variance (ANOVA).

### ELISA for the determination of IL-13, IL-22, TARC and AKG levels

Mouse sera were extracted by centrifuging whole blood samples at 8,000 × g for 5 min at room temperature. Serum concentrations of total IL-13, IL-22, TARC and AKG were quantified using the following ELISA kits, in accordance with the manufactures’ protocols: IL-13 (MULTI SCIENCES EK213), IL-22 (MULTI SCIENCES EK213), TARC (FineTest^®^ EM1388) and AKG (Cell Biolabs, INC, MET-5131). All experiments were independently repeated two to three times.

### Liquid chromatography mass spectrometry (LC-MS/MS)-tandem mass spectrometry analysis for BALB/c mice’s fecal and bacteria culture supernatant

To investigate anti-AD metabolites in BX-BC08, 100 µL of each liquid sample (BX-BC08 or BHI) was mixed with 400 µL of 80% methanol solution, while 100 mg of each fecal sample from BX-BC08 treated mice or BHI-treated mice was suspended in 500 µL of 80% methanol solution. Following the first centrifugation, the supernatant was diluted to 53% methanol and then centrifuged again for LC-MS analysis. Ultra-high performance liquid chromatography (UHPLC) separation was performed using a Vanquish UHPLC system (Thermo Fisher, Germany), equipped with a Hypesil Gold column (100 × 2.1 mm, 1.9 μm). The elution gradient is shown in Table S2. And Q Exactive™ HF/Q Exactive™ HF-X (Thermo Fisher, Germany) was used to acquire tandem mass spectrometry (MS/MS) spectra on an information-dependent basis during LC-MS/MS experiment. Raw LC-MS/MS data were processed using Compound Discoverer (V.3.3). Data analysis was performed using the Linux-based CentOS (V.6.6) operating system, as well as R and Python. Metabolite identification was conducted using the KEGG PATHWAY database (KEGG, genome.jp/kegg/pathway.html), the Human Metabolome Database (HMDB, www.hmdb.ca), and LIPID Maps (lipidmaps.org) for enhanced reliability. Metabolites specifically enriched in BX-BC08 compared to BHI, with an adjusted *P*-value < 0.05, were considered statistically significant.

### Cell culture

To determine whether AKG directly modulates the skin barrier function of keratinocytes, normal human epidermal keratinocytes (NHEKs; PCS-200-010, ATCC) were treated with recombinant human IL-4 (20 ng/ml; 574002, BioLegend) and IL-13 (10 ng/ml; 571102, BioLegend), with or without AKG, for 24 h. Following treatment, RNA was extracted to assess the expression of genes associated with skin barrier function.

### Flow cytometry analysis

To investigate whether AKG directly modulates T-cell polarization, we isolated T cells from mouse spleens using the magnetic cell separation method (EasySep™ Mouse T Cell Isolation Kit; STEMCELL Technologies). The isolated T cells were cultured in RPMI1640 medium supplemented with 10% FBS, 10ng/mL mouse recombinant IL-4, and 5 ng/ml IL-2. The cells were activated with Dynabeads mouse T-Activator CD3/CD28 beads (Thermo Fisher Scientific, Waltham, MA) for 72 h. After activation, all cells were collected for flow cytometry analysis.

### Western blot

Proteins were extracted using RIPA buffer (Pierce, Rockford, IL) on ice following tissue disruption with the Tissuelyser-64 L homogenizer (Shanghai Jingxin). Protein concentrations were determined using the BCA assay (FD2001, Hangzhou Fude Biology). Equal amounts of protein lysates were resolved on 4-20% SDS-PAGE polyacrylamide gels and transferred to PVDF membranes (0.22 μm, Millipore) via wet electrotransfer. Membranes were blocked with 5% Skimmed milk powder for 1 h at room temperature. After that, the membranes were incubated at 4 °C overnight with the following primary antibodies respectively: H3 (#17168-1-AP, proteintech, dilution ratio 1:2000), H3K9me3 (#M1112-3, HUABIO, dilution ratio 1:2000), and H3K27me3 (#HA722231, HUABIO, dilution ratio 1:2000). On the subsequent day, the blots were subjected to an incubation process with horseradish peroxidase (HRP)-conjugated secondary antibodies (#RM3002, Beijing Ray Antibody Biotech, with a dilution ratio of 1:5000) at room temperature for a duration of 1 h. Detection was performed using enhanced chemiluminescence (ECL, Millipore).

### Statistical analysis

All experimental data were presented as mean ± standard error of the mean (S.E.M) for both in vivo and in vitro investigations. Intergroup comparisons were analyzed using independent two-tailed Student’s *t*-test with Welch’s correction for unequal variances (Altman, 1990). Multi-group comparisons were conducted through ANOVA followed by Tukey’s post hoc test for pairwise comparisons (Armstrong, Eperjesi, & Gilmartin, 2002). Statistical significance was defined as *p* < 0.05, with exact *p*-values reported for all analyses in accordance with contemporary statistical reporting guidelines (Wasserstein & Lazar, 2016).

## Results

### *B. lactis* BX-BC08 alleviates MC903-induced AD-like inflammation in BALB/c mice

**Fig. 1:**
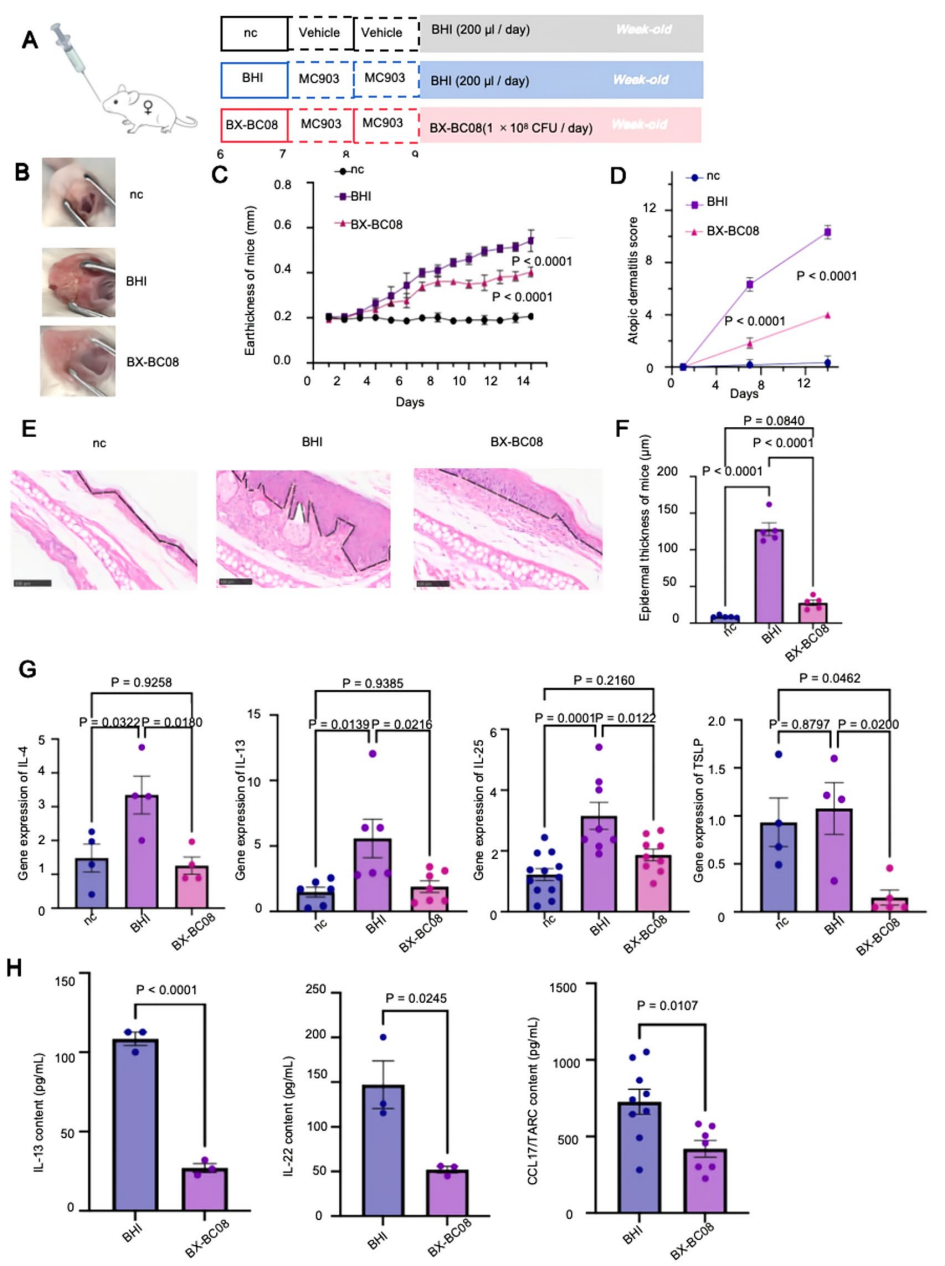
*B. lactis* BX-BC08 alleviates MC903-induced AD-like skin inflammation in BALB/c mice. (**A**) Schematic diagram illustrating the experimental design and timeline of the female MC903 mice model. (**B**) Representative ear pictures at day 14. (**C**) Ear thickness measurements taken from day 0 to day 14. (**D**) Temporal pattern of AD scores across different treatment regimens. (**E**) Representative ear sections were stained with H&E for the indicated groups. Scale bar = 20 x, 100 μm. The black line delineates the basement membrane. (**F**) Quantification of the ear epidermal thickness from panel (**E**). (**G**) Gene expression levels of IL-4, IL-13, IL-25, and TSLP in the ear from each group. (**H**) Measurement of serum cytokine levels (IL13, IL22 and CCL17/TRAC) in mice for the indicated treatments. All data represent two to three independent experiments and are presented as means ± S.E.M. Significant differences between groups were determined using ANOVA

To evaluate the preventive effects of *Bifidobacterium* strain BX-BC08 on AD progression, we utilized a MC903-induced AD-like inflammation model in 6-week-old female BALB/c mice. Mice were gavaged once daily for three weeks with either *B. lactis* BX-BC08 (1 × 10^8^ CFU per mouse), BHI broth alone was used as a vehicle control to exclude any broth-related effects and establish a baseline control (Fig. 1A).

After one week of BX-BC08 administration, MC903, well-established inducer of type 2 skin inflammation that closely mimics human AD-like pathology, was applied daily to the ears. Inflammatory responses were evaluated in a blinded fashion. Ear thickness was measured using a digital caliper by investigators blinded to treatment group allocation. BX-BC08-treated mice exhibited significantly lower AD conditions on day 14 (Fig. 1B) from the naked eyes and reduced ear thickness starting at day 8 compared to BHI-treated mice (Fig. 1C). A modified AD severity score was independently evaluated by two trained observers, both blinded to treatment group assignments. At the study endpoint, BX-BC08-treated mice got lower AD scores (Fig. 1D) and displayed less swelling than BHI-treated mice on the epidermal thickness (Fig. 1E). Histological analysis further revealed that BX-BC08 treatment mitigated MC903-induced epidermal hyperplasia and inflammatory cell infiltration (Fig. 1F). To explore the underlying immunological mechanisms in both local and systemic effects, we assessed the gene expression of key Th2 cytokines (IL-4, IL-13) [20], the early inflammatory driver IL-25 [21], and TSLP, a keratinocyte-derived factor linked to epidermal hyperplasia [18] in ear tissue. BX-BC08-treated mice exhibited reduced expression of these cytokines compared to BHI-treated mice (Fig. 1G). In addition to its local effects, BX-BC08 exerted systemic anti-inflammatory activity, as evidenced by reduced serum levels of Th2-associated cytokines IL-13 and TARC/CCL17, as well as the T helper 17 (Th17)-related cytokine IL-22 (Fig. 1H). These findings suggest that *B. lactis* BX-BC08 alleviates MC903-induced AD-like inflammation, potentially through the suppression of inflammatory cytokine expression at both local and systemic levels.

### *B. lactis* BX-BC08 alters the gut microbiota with enriched probiotics in MC903-treated mice

**Fig. 2:**
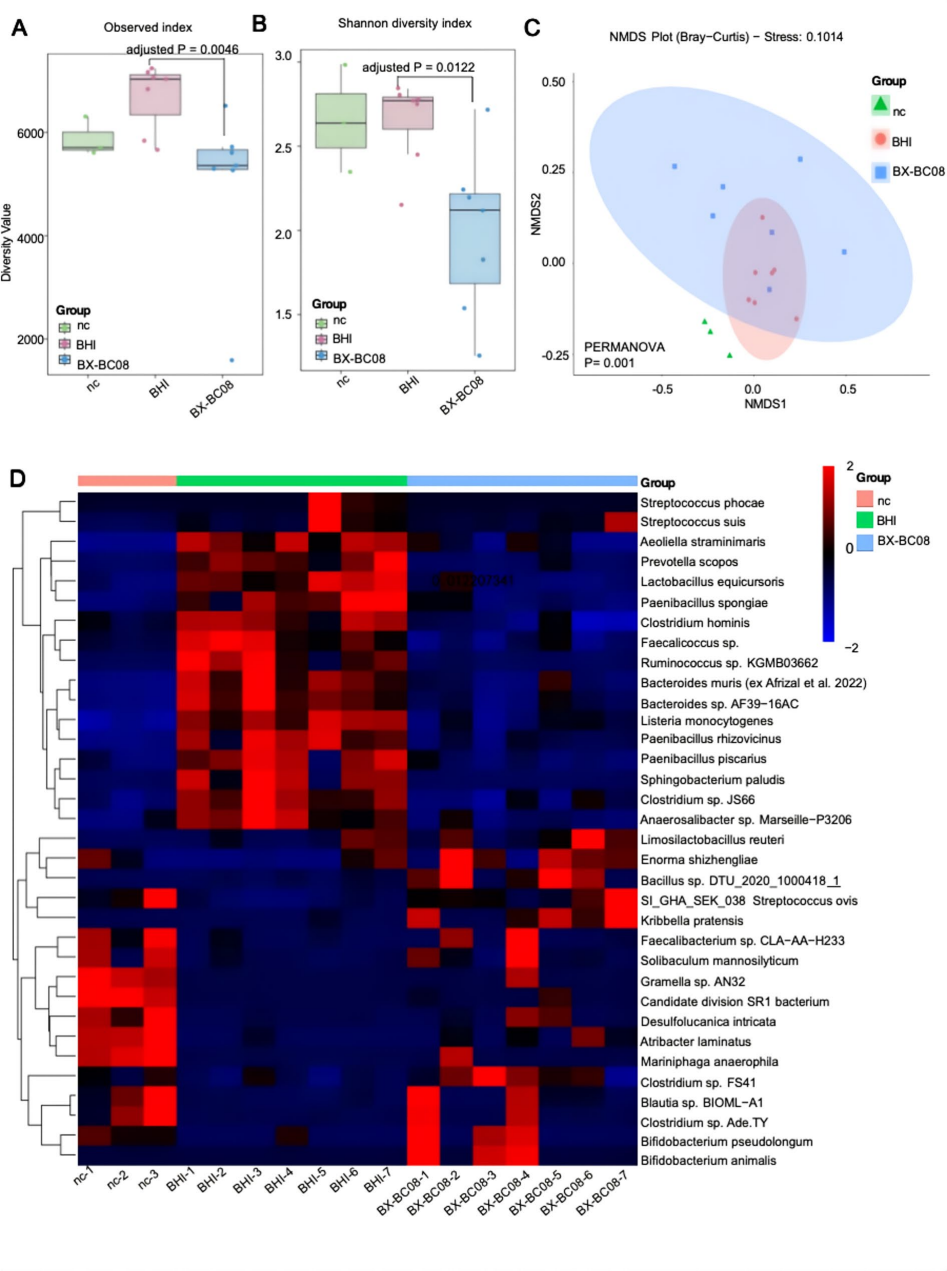
BX-BC08 modulates the gut microbiota of MC903-induced AD mice. (**A**) Alpha- diversity analysis of gut microbiota in observed richness and (**B**) Shannon index. (**C**) The NMDS plot shows that there are significant differences in the microbial communities of different groups. (**D**) Heatmap displaying differentially abundant bacterial OTUs (adjusted *P* < 0.05). Each row corresponds to a bacterial genus, and each column represents an individual mouse sample. The color scale reflects the log_2_-transformed relative abundance of each genus. *P* values were adjusted using the Benjamini-Hochberg (BH) procedure to control for false discovery rate

To evaluate the impact of *B. lactis* BX-BC08 on the gut microbiota, we performed metagenomic sequencing on stool samples from MC903-treated mice following three weeks of oral administration with either water (nc), BHI or BX-BC08. BX-BC08 treatment did significantly alter species richness, as indicated by the observed index (Fig. 2A). When considering both species richness and evenness (Shannon diversity index), BX-BC08 also significantly influenced the relative abundance of gut microbial species (*P* = 0.0122) (Fig. 2B). The NMDS plot, based on Bray-Curtis distances, illustrates the similarity of microbial communities among samples. A statistically significant separation was observed among the groups (*P* = 0.001), indicating distinct differences in microbial composition (Fig. 2C). We also observed the BX-BC08 group and the nc group shared certain similarities in the regulation of intestinal microbiota composition. Notably, BX-BC08 administration increased the relative abundance of well-characterized symbiotic probiotic families, including *Bifidobacteriaceae* and *Lactobacillaceae*, which are known to directly support anti-inflammatory responses and skin barrier function (O’Neill, Monteleone, McLaughlin, & Paus [22]). Conversely, compared with the BHI group, the BX-BC08 group exhibited a significant reduction in bacterial genera associated with pro-inflammatory activity, such as *Streptococcus suis* (Fig. 2D). These findings suggest that *B. lactis* BX-BC08 modulates the gut microbiota by promoting beneficial bacteria while reducing potentially pro-inflammatory taxa in MC903-treated mice.

### *B. lactis* BX-BC08 produces alpha-Ketoglutaric acid in supernatant and MC903-treatd mice

**Fig. 3:**
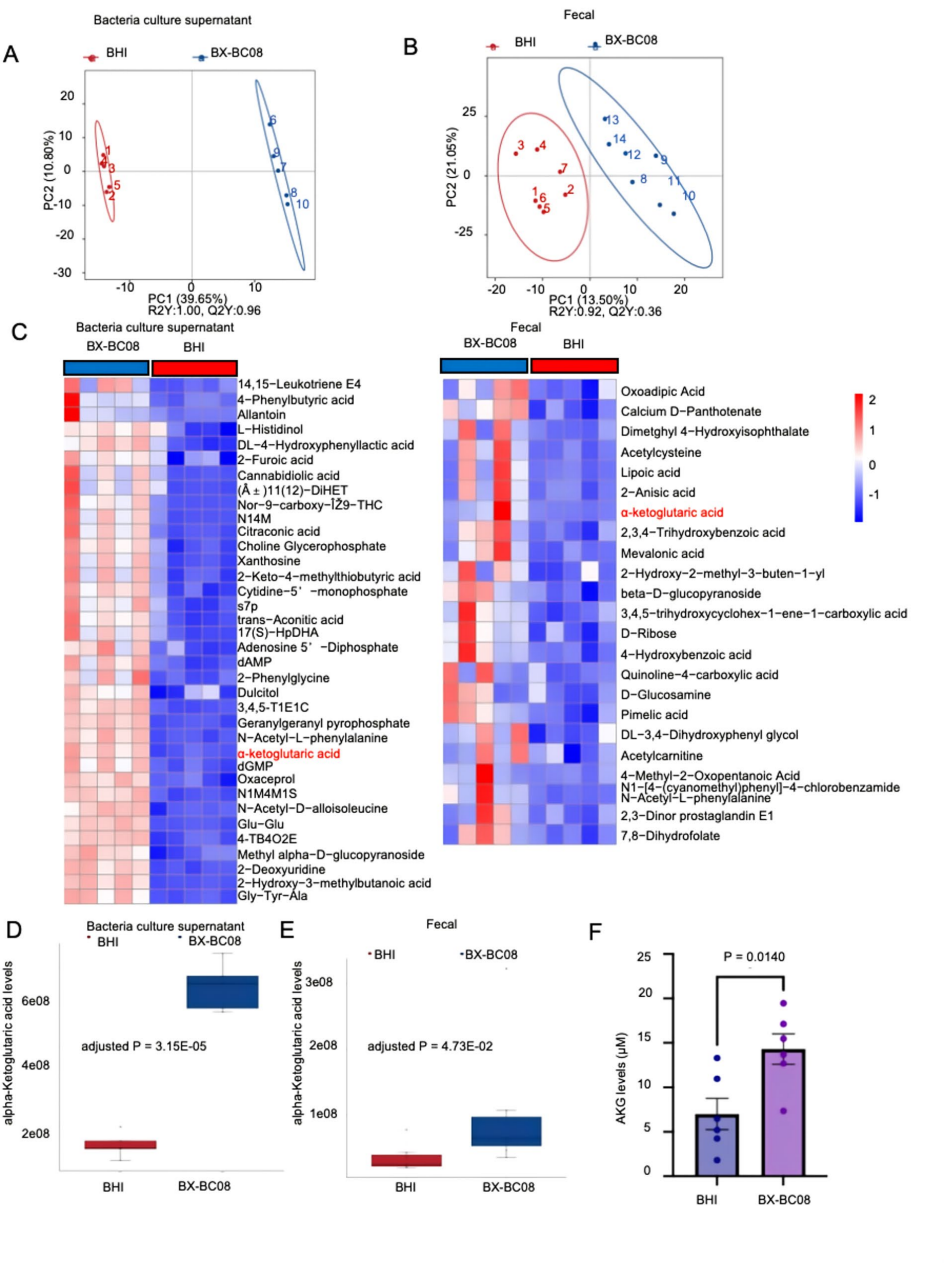
Metabolic profiling of fecal samples and *B. lactis* BX-BC08 supernatant in BALB/c mice with AD. (**A**) PCA score plot illustrating the clustering of bacteria culture supernatant from BHI and BX-BC08. The PCA plot demonstrates a distinct separation between the two groups, suggesting differences in their metabolic profiles. Principal component 1 (PC1) accounts for 39.65% of the variance, while principal component 2 (PC2) explains 10.80%. (**B**) PCA score plot illustrating the clustering of fecal samples from BHI-treated mice and BX-BC08-treated mice. PC1 accounts for 13.50% of the variance, while PC2 explains 21.05%. (**C**) Heatmap showing the relative abundance of metabolites in bacteria culture supernatant or fecal samples from BALB/c mice treated with BHI or BX-BC08. Each row corresponds to a metabolite, while each column represents an individual mouse. The color scale reflects the log₂-transformed relative abundance of each metabolite, allowing for a comparative assessment of metabolic variations across samples. (**D**) Box plots illustrating the quantitative levels of AKG in bacteria culture supernatant from BHI and BX-BC08. (**E**) Box plots displaying the quantitative levels of AKG in fecal samples from BALB/c mice treated with BHI or BX-BC08. (**F**) The serum level of AKG in BHI group and BX-BC08-treated group. *P* values are calculated by one-way analysis of variance. We use abbreviations for some metabolites in the heatmap: N1-(3-cyano-4,6-diphenyl-2-pyridyl) -4-methylbenzamide: N14M, 3,4,5-trihydroxycyclohex-1-ene-1-carboxylic acid: 3,4,5-T1E1C, N1-morpholinocarbonyl-4-methylbenzene-1-sulfonamide: N1M4M1S, 4- [4-(tert-butoxy carbonyl) piperazino] -4-oxobut-2-enoicacid: 4-TB4O2E. *P* values for panels D and E were adjusted using the BH procedure to control the false discovery rate

To investigate the specific anti-inflammatory molecules produced by *B. lactis* BX-BC08, we conducted non-targeted metabolomics sequencing on bacteria culture supernatant and fecal samples from BX-BC08-treated mice. Principal component analysis revealed significant differences in metabolite profiles between the BX-BC08 and BHI control groups, both in bacteria culture supernatant (Fig. 3A) and fecal samples (Fig. 3B) from BX-BC08-treated mice in the MC903-induced murine model.

Further differential abundance analysis of both bacteria culture supernatant and fecal samples (Fig. 3C) identified AKG as the only metabolite produced by *B. lactis* BX-BC08, distinct from metabolites derived from host metabolism. This finding was further supported by the relative quantitative data, showing a more than three-fold enrichment of AKG in the bacteria culture supernatant (Fig. 3D), as compared to fecal samples from BHI-treated mice (Fig. 3E). To determine whether BX-BC08 produces AKG and contributes to its systemic availability, we measured serum AKG levels. Compared to BHI-treated controls, mice receiving BX-BC08 showed a significant elevation in serum AKG concentrations (Fig. 3F), suggesting that AKG is a key mediator exerted by BX-BC08. These results indicate that *B. lactis* BX-BC08 produces AKG, which may act as a bioactive metabolite with potential therapeutic implications for alleviating AD.

### alpha-Ketoglutaric acid ameliorates MC903-induced AD-like inflammation in BALB/c mice

**Fig. 4:**
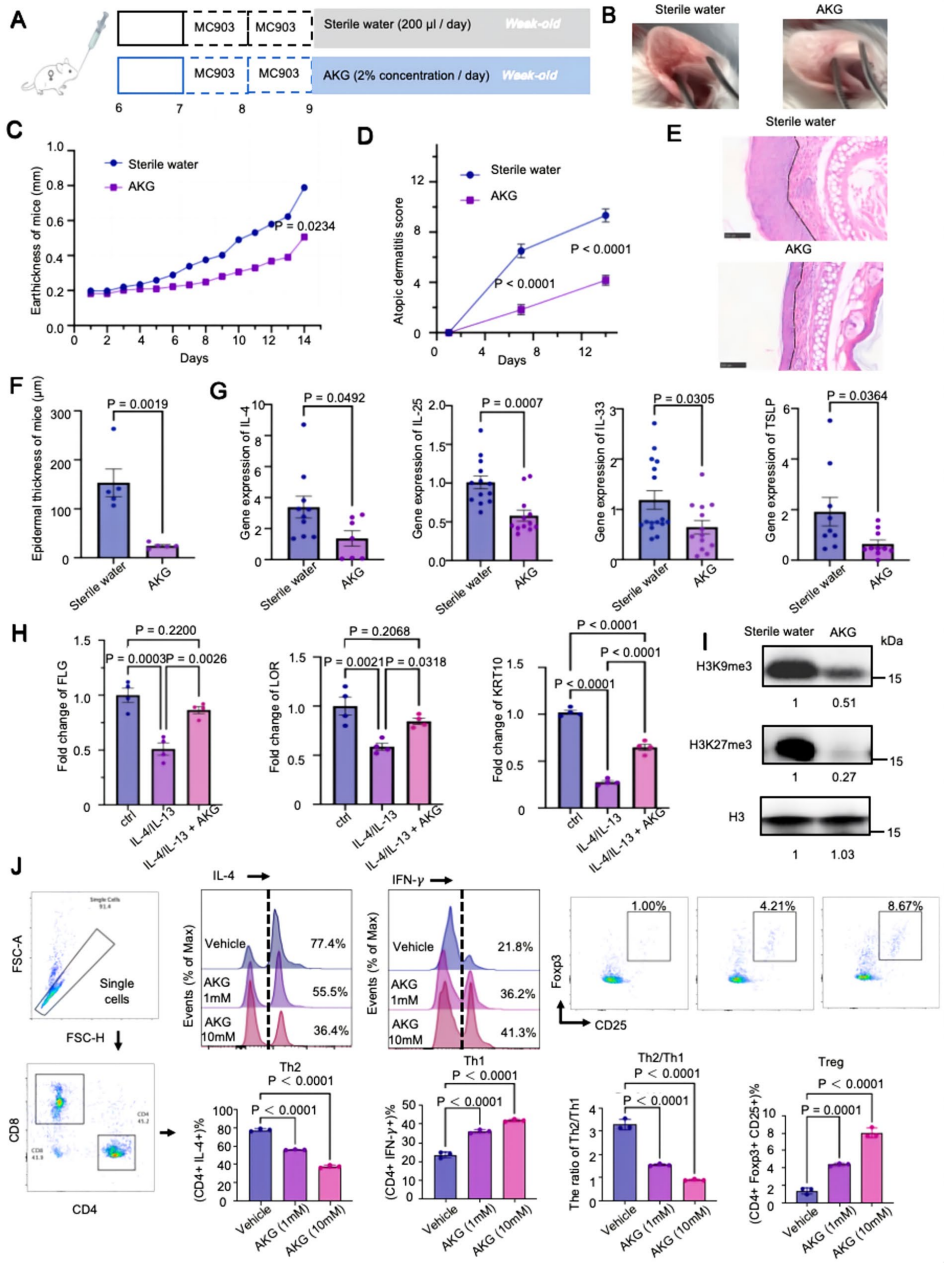
AKG reduced MC903-induced AD-like skin inflammation in BALB/c mice. (**A**) Schematic diagram illustrating the experimental design and timeline of the female MC903 mice model. (**B**) Representative ear pictures at day 14. (**C**) Ear thickness measurements taken from day 0 to day 14 with or without AKG treatment. (**D**) Temporal pattern of AD scores across with or without AKG treatment. (**E**) Representative ear sections were stained with H&E for the indicated groups. Scale bar = 20 ×, 100 μm. The black line delineates the basement membrane. (**F**) Quantification of the ear epidermal thickness from panel (**E**). (**G**) Gene expression levels of IL-4, IL-25, IL-33, and TSLP in the ear from the indicated group. (**H**) Gene expression levels of FLG, LOR, and KRT10 in IL-4/IL-13-exposed keratinocytes, with or without AKG treatment. (**I**) Western blot analysis of H3K9me3 and H3K27me3 in ear tissues was performed for both the sterile water group and the AKG-treated group. (**J**) Effects of the indicated concentrations of AKG on T cell polarization. All data represent two to three independent experiments and are presented as means ± S.E.M. Significant differences between two groups were assessed using an unpaired Student’s *t*-test, while comparisons among three or more groups were performed using one-way ANOVA

To further investigate whether AKG produced by *B. lactis* BX-BC08 contributes to the alleviation of AD-like inflammation, BALB/c mice were administered a 2% AKG solution prior to MC903-induced ear inflammation. After seven days of AKG treatment, MC903 was applied to the inner surfaces of both ears to induce AD-like inflammation (Fig. 4A). Assessments were conducted in a blinded manner: erythema, swelling, and scaling were independently scored by two trained observers blinded to treatment allocation (sterile water vs. AKG), while ear thickness was measured with a digital caliper by operators unaware of group assignments. Mice treated with AKG displayed less erythema, swelling, and scaling than the sterile water-treated group (Fig. 4B), as well as a reduction in ear thickness (*P* = 0.0234; Fig. 4C). By day 14, blinded assessment of the modified AD severity index demonstrated a significant reduction score in AKG-treated mice (Fig. 4D). Histological analysis further confirmed that AKG treatment mitigated MC903-induced epidermal hyperplasia and inflammatory cell infiltrations in sterile water-treated mice (Fig. 4E-F). Consistent with these observations, gene expression analysis revealed that AKG treatment suppressed the expression of AD-associated pro-inflammatory cytokines, including IL-4, IL-25, IL-33, and TSLP (Fig. 4G). Given the close association between epidermal hyperplasia and skin barrier integrity, we next investigated the direct effects of AKG on skin barrier function. To induce barrier dysfunction, normal human epidermal keratinocytes were stimulated with recombinant IL-4 and IL-13. AKG treatment reversed IL-4/IL-13- induced skin barrier dysfunction by upregulating key differentiation markers, including FLG, LOR, and KRT10 (Fig. 4H). Western blot analysis further revealed that AKG supplementation significantly reduced the levels of repressive histone modifications H3K9me3 and H3K27me3 (Fig. 4I), suggesting that AKG may exert its effects, at least in part, through epigenetic mechanisms such as DNA demethylation [23]. In parallel, AKG treatment led to a reduction in IL4 expression. IL-4, a hallmark Th2 cytokine, promoted Th2 differentiation and suppressed T helper 1 (Th1) responses. To further explore the immunomodulatory effects of AKG, we assessed its impact on T cell polarization by using isolated naïve T cells. AKG exhibited a dose-dependent suppression of Th2 cell differentiation, while enhancing the proportions of Th1 and regulatory T (Treg) cells (Fig. 4J). Collectively, these findings provide compelling evidence that AKG, a metabolic product of *B. lactis* BX-BC08, holds therapeutic potential for ameliorating type 2 inflammation at least in part by restoring skin barrier function and rebalancing between Th2/Th1 ratio as well as Treg responses.

## Discussion

In this study, we provide the evidence that oral administration of BX-BC08 reduced AD-like inflammation and epidermal thickening in a MC903-induced AD model. Epidemiological studies and preclinical experiments have shown that certain probiotics can mitigate the incidence of AD [24]; Frei, Akdis, & O’Mahony [25]; Rautava, Kainonen, Salminen, & Isolauri [26]). For example, oral administration of *Bifidobacterium breve* M-16 V and *Bifidobacterium longum* BB536 has been reported to lower AD incidence compared to controls [27]. Additionally, a study demonstrated that a six-month treatment with a probiotic cocktail containing *Lactobacillus* and *Bifidobacterium* significantly reduce the SCORAD index [28]. However, the underlying mechanisms by which probiotics influence AD remain unclear.

We conducted a metagenomics analysis and found that BX-BC08 significantly increased the abundance of well-characterized commensal probiotics, such as *S.hyointestinalis* and *L. reuteri*, while depleting potential pro-inflammatory pathogenic species, including *S. phocae and S.suis.* Notably, *L. reuteri* is known to suppress inflammation by enhancing the production of immunosuppressive mediators, TGF-β and IL-10 (Huang, Shi, Yang, & Wang [29]; Kiššová, Tkáčiková, Mudroňová, & Bhide [30]). In addition, BX-BC08 enhanced the relative abundance of the families *Bifidobacteriaceae* and *Lactobacillaceae*. Extracellular vesicles derived from *Bifidobacteriaceae* have been reported to improve skin barrier integrity [31], and oral supplementation with *Lactobacillus acidophilus* has been shown to alleviate skin dryness and reduce transepidermal water loss [32]. In contrast, *S. suis* triggers the release of pro-inflammatory cytokines and chemokines in macrophages [33]. Additionally, previous studies have demonstrated that fecal microbiota transplantation can ameliorate AD in mice [16], and probiotics have been shown to alter gut microbiota composition to restore Th1/Th2 balance (Tan, Zhu, Du, Zhang, & Yin [34]). These findings suggest that BX-BC08 alleviates AD-like inflammation, at least in part, by enriching beneficial probiotics and reducing the abundance of potential pro-inflammatory pathogens.

We also demonstrated that metabolites produced by BX-BC08 can mitigate Th2-driven immune responses. Metabolomic analysis revealed that BX-BC08 produces AKG, a metabolite known to enhance dermal integrity and strengthen the epidermal barrier (Gyanwali et al., 2022) (Dobrowolski, Tomaszewska, Bienko, Radzki, & Pierzynowski [35]). Beyond its role in skin barrier protection, gut microbiota derived AKG has been reported to inhibit immune cell activation and decrease the production of pro-inflammatory cytokines [36]. On this notion, AKG plays a pivotal role in detoxifying reactive oxygen species (ROS) and influencing DNA demethylation [37]; Tran, Dillingham, & Sridharan [38]; Yang, Zhou, Guo, & Zhou [39]), (Meng, Liu, Peng, He, & Li [40]). Notably, inhibiting ROS has been shown to suppress the spontaneous hyperexpression of IL-4, a hallmark of AD-associated inflammation [41]. Furthermore, DNA demethylation contributes to regulatory T cell differentiation and anti-inflammatory macrophage populations. In addition, AKG can directly activate T cells to stimulate IL-10 production, thereby contributing to the suppression of chronic inflammation [42]. Beyond its immunomodulatory effects on macrophages and T cell subsets, AKG has been shown to promote beige adipocyte differentiation [43], thereby enhancing the anti-inflammatory and neuroprotective capacities of subcutaneous adipose tissue [44]. Thus, the anti-inflammatory properties of BX-BC08 may partially be attributed to its production of protective metabolites. However, further investigation is needed to determine whether AKG is the primary metabolite responsible for suppressing AD-like inflammation.

For long-term use, AKG has been shown to reduce systemic inflammatory cytokines and extend health span in animal models without causing significant side effects [42]. Clinically, AKG has been evaluated in several studies, all of which reported no adverse reactions (Riedel, Nündel, & Hampl [45]) [46], and confirmed its stability for intravenous administration (Wernerman, Hammarqvist, & Vinnars [47]). Collectively, these findings support the long-term safety and therapeutic potential of AKG in clinical settings. Our work further expands on these findings by highlighting the potential of AKG in the treatment of AD.

To our knowledge, this study provides the mechanistic evidence that AKG alleviates AD by modulating the gut microbiota. These findings introduce a novel therapeutic strategy that harnesses probiotic-derived metabolites for AD management and underscore the potential of combinatorial bacteriotherapy to restore gut-skin axis homeostasis.

This study has certain limitations. Further research is needed to determine whether dietary supplementation with AKG can alleviate AD through ectopic induction in vivo. Additionally, while our data demonstrates that AKG reduces AD severity in female mice, the underlying mechanisms have not yet been investigated in male animals.

## Electronic supplementary material

Below is the link to the electronic supplementary material.

Supplementary Material 1

Supplementary Material 2

## Data Availability

All data and code necessary to evaluate the conclusions of this study are provided in the main text. Metagenomic and metabolomic datasets are included in Supplementary Data 1. Additional datasets are available upon request from the lead contact.
