# Supplementary material for: Bifidobacterium animalis subsp. Lactis BX-BC08 modulates gut microbiota and secretes alpha-Ketoglutaric acid to alleviate MC903-induced atopic dermatitis
Source: J Transl Med. 2025 Jul 10;23:768. doi: 10.1186/s12967-025-06769-9 (PMC12247329; doi:10.1186/s12967-025-06769-9)
Supplement: Supplementary file 2 — Supplementary Material 2 [file 12967_2025_6769_MOESM2_ESM.docx]

**Table S1. Primers for Real-Time PCR analysis**

| Primer name | Sequence (5’ – 3’) | |
| --- | --- | --- |
|  | Forward Primer | Reverse Primer |
| Mouse IL-4 | GGTCTCAACCCCCAGCTAGT | GCCGATGATCTCTCTCAAGTGAT |
| Mouse IL-13 | CCTGGCTCTTGCTTGCCTT | GGTCTTGTGTGATGTTGCTCA |
| Mouse IL-25 | ACAGGGACTTGAATCGGGTC | TGGTAAAGTGGGACGGAGTTG |
| Mouse IL-33 | TCCAACTCCAAGATTTCCCCG | CATGCAGTAGACATGGCAGAA |
| Mouse TSLP | ACGGATGGGGCTAACTTACAA | AGTCCTCGATTTGCTCGAACT |
| Human FLG | GCTGAAGGAACTTCTGGAAAAGG | GTTGTGGTCTATATCCAAGTGATC |
| Human LOR | GTCTGCGGAGGTGGTTCCTCT | TGCTGGGTCTGGTGGCAGATC |
| Human KRT10 | CCTGCTTCAGATCGACAATGCC | ATCTCCAGGTCAGCCTTGGTCA |

**Table S2. Chromatographic gradient elution procedure**

| Time (mins) | A (0.1% formic acid) % | B (methyl alcohol) % |
| --- | --- | --- |
| 0 | 98 | 2 |
| 1.5 | 98 | 2 |
| 3 | 15 | 85 |
| 10 | 0 | 100 |
| 10.1 | 98 | 2 |
| 11 | 98 | 2 |
| 12 | 98 | 2 |
